# Improving Vehicle Connectivity Through a Novel Self-Organizing Network Mechanism

**DOI:** 10.3390/s25196037

**Published:** 2025-10-01

**Authors:** Chia-Sheng Tsai, Chia-Kai Wen

**Affiliations:** Department of Computer Science and Engineering, Tatung University, Taipei 104, Taiwan

**Keywords:** V2V communications, self-organized to clusters, LiDAR sensors

## Abstract

A trend analysis mentioned that the global automotive Vehicle-to-Everything—also called V2X—market size will be reached at several billions in the near future. This information clearly highlights the importance of developing V2X communication. Nowadays, automobile manufacturers have introduced vehicles equipped with driver assistance and even conditional autonomous driving features. Light detection and ranging (LiDAR) components are used in sensor networks to detect objects around. Also, vehicles take advantage of LiDAR sensors to discover the neighbor cars in cognitive systems for road safety. Carrying on from our previous works, we found that organizing vehicles into groups can enhance the safety of the vehicle networks by LiDAR assistance. However, the success rate and reliability of grouping vehicles is an important issue. Also, enhancing existing Vehicle-to-Vehicle (V2V) communication mechanisms remains a key factor in ensuring that emergency messages can be transmitted both timely and accurately. To address this, in this research, a method is proposed to make vehicles on the road be self-organized well for Intelligent Transportation Systems (ITS). Also, we found that before data in each car is transmitted, the scenario that data is queued for waiting a random time exponentially distributed outperforms data being sent immediately.

## 1. Introduction

Vehicle-to-Everything (V2X) is expected to reach USD 66.26 billion by 2032; the compound annual growth rate (CAGR) during the forecast period from 2024 to 2032 should be hit 39.46% [1]. Regarding V2X systems research, most authors in the literature studied it by using simulation tools such as Network Simulation-2 (NS2), Graphical Network Simulator-3 (GNS3), Eclipse Simulation of Urban Mobility (SUMO), and so on. In this paper, we use the programming language C and shell script for implementation to enhance elasticity and adaptability. First, relevant state-of-the-art standards and specifications are studied to ensure no violation in the proposed algorithm. Secondly, V2X articles in the literature are comprehensively read and summarized in the appendix, leading to the proposed mechanism suitable to most V2X environments. Finally, the detailed steps of the proposed schema, numerical results, and concluding remarks are also described in this paper.

**Figure 1:**
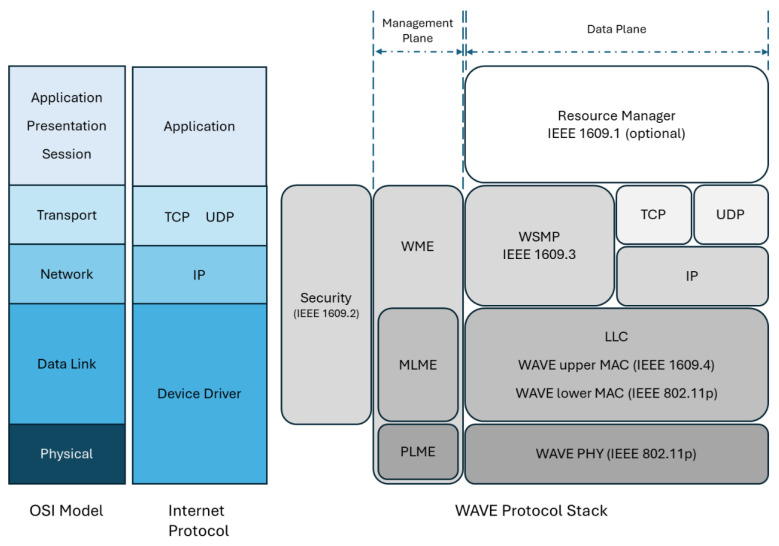
The related mapping layer for WAVE and OSI.

**Figure 2:**
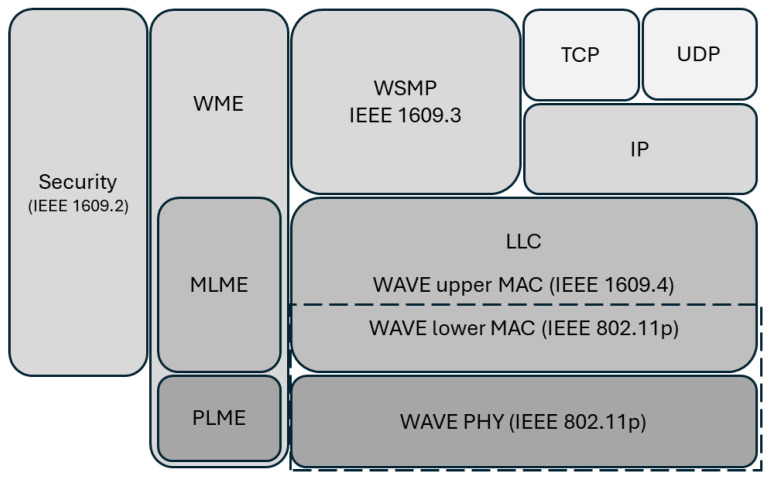
Scope of IEEE 802.11p in the dashed box.

According to a United Nations (UN) report [2], road traffic accidents continue to be the leading killer of children, youth, and the elderly, resulting in several deaths daily. Such incidents are posing severe and increasing mortality risks to pedestrians, cyclists, and other vulnerable road users. Hence, a method to strengthen the connectivity of vehicles to send warning messages to each other is crucial. To continue our previous research [3,4], we studied the mechanism of cooperation of vehicles nowadays. First of all, we surveyed and summarized the relevant state-of-the-art standards and specifications in Figure 1 and Figure 2. To prove that the scheme we proposed is feasible and fulfills the protocol requirement, we collected and described the relevant research in Section 2.

With the development of wireless networks and the rise of V2X, a new concept of transportation has emerged for humanity. V2X—vehicle-to-everything—refers to the interconnection of vehicles with everything around them, primarily including V2P (vehicle-to-pedestrian), V2V (vehicle-to-vehicle), V2I (vehicle-to-infrastructure), and V2N (vehicle-to-network). In general, vehicles are equipped LiDAR sensors to discover neighboring cars in cognitive systems for traffic safety [5,6,7,8,9,10,11,12]. By connecting and exchanging information between vehicles and pedestrians, other vehicles, and infrastructures, and utilizing cloud computing to share traffic information, V2X enhances transportation safety and driving efficiency, and even promotes energy conservation and carbon reduction. Furthermore, it provides a smart and sustainable development of transportation. For brevity and convenience, we have listed commonly used terms in Table 1 clearly.

Currently, the mainstream communication technologies for V2X are divided into DSRC (Dedicated Short-Range Communication) and C-V2X (Cellular Vehicle-to-Everything) [13,14,15,16]. The former is a mature technology suitable for real-time communication, while the latter leverages existing cellular network infrastructure to provide great application scalability and flexibility. The two communication methods have their own advantages and can be applied according to local conditions. For example, in areas lacking fifth-generation system (5G) infrastructure, DSRC can be considered, whereas in regions with established 5G networks, DSRC and C-V2X both can be considered simultaneously.

In the world of V2X, DSRC is a service used for V2V and V2I communication. It enhances public driving safety and can also be used for private or commercial purposes outside of public utilities. In October 1999, the United States Federal Communications Commission (U.S. FCC) allocated the 5.9 GHz band for ITS applications based on DSRC and passed the basic technical rules for DSRC operation [13]. Currently, this band is divided into seven channels, each 10 MHz wide. The first and last channels are reserved solely for safety purposes.

The service based on the wireless communication technology is known as WAVE (Wireless Access in Vehicular Environment), IEEE standards that include IEEE 1609 standards [17] and the IEEE 802.11p communication protocol. As shown in Figure 1, the WAVE protocol stack can be divided into two parts: the left half pertains to the management plane, while the right half pertains to the data plane. When mapping the stack to the OSI model, it can be observed that IEEE 1609.1 standard corresponds to the application layer, IEEE 1609.2 spans from the data link to the transport layer, IEEE 1609.3 covers the network and transport layers, IEEE 1609.4 is at the data link layer, IEEE 802.11p corresponds to the physical and data link layers. WAVE supports IP-based and non-IP-based data transmission, but individual devices might support only one networking stack.

IEEE 1609 is a standard for V2V and V2I communication; the primary difference from general network applications lies in the characteristics of vehicle movement. During the rapid movement of vehicles, the characteristics of time and location should be considered in security mechanisms to provide comprehensive services within an appropriate time, ensuring the information security requirements of the Internet of Vehicles (IoV). The packet transmission length in IoV communication is limited, necessitating the use of shorter messages to transmit and exchange data (As IEEE 1609.3—WAVE short message protocol (WSMP) intends). Due to the advantage of ECC (Elliptic Curve Cryptography), with its shorter key length and certain level of security, the IEEE 1609.2 standard employs ECC-based signing and encryption methods to ensure information security.

In Figure 2, the work of IEEE 802.11p includes the medium access control (MAC) and physical (PHY) layers. It is known that the DSRC spectrum is divided into one control channel (CCH) and six service channel (SCHs) and has short delay transmission characteristics, and these specific channels in application scenarios will be provided to multiple users for access. Therefore, different access categories and different priority management methods need to be considered to avoid channel preemption problems.

To this end, establishing a transportation system based on V2X communication is one of the development priorities for advanced countries (such as European countries, the United States, and Japan). In recent years, the flourishing development of the Internet of Things (IoT) and wireless communication technologies has enabled the Internet of Vehicles to utilize various communication technologies, such as Cellular Vehicle-to-Everything (C-V2X), which is defined by third-generation partnership project (3GPP) standards [18], to integrate information and provide real-time and diverse services.

Taking the development of ITS in Europe as an example, the Car-to-Car Communication Consortium (C2C-CC) is an organization which was established by automobile manufacturers in 2002 [19] to standardize wireless communication between vehicles and the environment. This allows vehicles from different manufacturers to work together and communicate with roadside units. The consortium members began developing a minimum set of interoperable cooperative intelligent transport systems (C-ITS) through the European Telecommunications Standards Institute (ETSI) and International Organization for Standardization (ISO) [20] standards in 2009. ETSI, as the organization responsible for standardizing the telecommunications industry, has established multiple standards. For example, it developed “TS 102 792”, a mitigation technique to avoid disturbance between European DSRC devices and ITS operating within the 5 GHz frequency range [21]. A decade later, the development of V2X reached an important milestone with the introduction of the first vehicles equipped with cooperative V2X in the European market [22]. ETSI ITS G5 technology is applied to these vehicles for V2X communication [23], including V2I communication capable of interacting with signal phase and timing (SPaT) [24]. And, in recent years, with the flourishing development of machine learning and artificial intelligence algorithms, coupled with the application of sensors and multi-access edge computing, it will be possible to introduce digital twin technology [25] in the future to optimize the operation strategy of traffic signals to improve the efficiency of transportation. So, this implies that, in response to the still busy traffic conditions in the future, low packet loss rates and low latency are the most important issues in vehicular ad hoc networks (VANETs).

**Figure 3:**
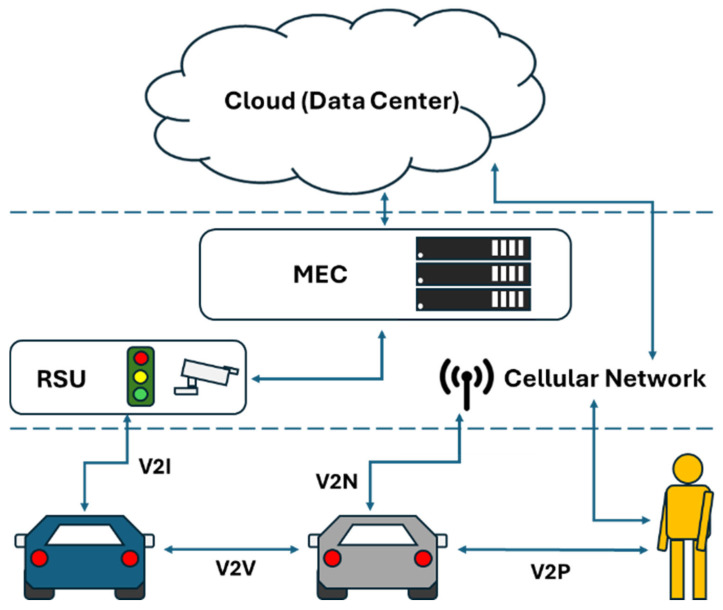
C-V2X communication environments. The arrows indicate communication between the different elements. The dashed lines represent separate stacks.

With the rise of connected vehicles and older-generation vehicles being intricately combined on the road, improving the transportation efficiency remains challenging. Due to vehicles being equipped with hardware devices with limited storage and computing capabilities, some tasks must be offloaded to mobile edge computing (MEC) to mitigate delay and security issues effectively. Thus, considering MEC-based C-V2X communication can cover most of urban traffic scenarios as shown in Figure 3.

To sum up, V2X has experienced exponential growth in market size over the past years. Carrying on from our previous research [3,4], we found that strengthening the connectivity of vehicles on a road conduces the collaboration of cars to exchange information with each other. Thus, several traffic situations can be improved, such as communication performance and road safety. For now, most traffic incidents are posing severe and increasing mortality risks to pedestrians, cyclists, and other vulnerable road users. In our past study [3,4], we proposed a method to send warning messages within elastic coverage to improve road safety. However, nowadays, LiDAR sensors are usually equipped in cars to detect objects around. Vehicles usually take advantage of LiDAR sensors to discover neighboring cars for traffic safety [5,6,7,8,9,10,11,12]. Therefore, in this paper, we proposed a self-organization scheme to group vehicles into clusters efficiently. The K-means algorithm is a way to solve this problem but the K value is difficult to be determined [26,27]. Hence, we need to consider a practical model to organize vehicles on a road successfully. Also, using the mechanism proposed here, the communication throughput is satisfied. In the study, two simulation scenarios were considered based on actual traffic conditions and we attempted to find the most effective configuration method. In order to fulfill many of the specifications for V2X requirements mentioned above, in this paper, the mechanism we proposed to organize vehicles into clusters successfully is feasible in most environments in practice.

## 2. Related Works

With the benefit of clustering, the packet routing stability of vehicle networks can be achieved, and road safety can be improved [3,4]. However, the two questions here are (1) Is the grouping concept suitable for most V2X systems? (2) Could the clustering algorithm be applied to time-limited V2X environments? To answer these two questions, first, we surveyed several studies on vehicle communication networks in the literature to prove that the scheme we designed is appropriate. Regarding vehicle networks, we collected miscellaneous papers in the literature for study. Secondly, the question is, is a clustering algorithm easy to be realized? Many methods are based on modified K-means approach for clustering. The authors of some articles mentioned that the K value is usually difficult to be determined in practice [26,28,29,30].

In [28], taking the K-means approach as an example, one technique known as “spectral clustering” transforms the clustering problem into a graph-cut problem in graph theory and applies the Laplacian matrix’s spectral properties (eigenvalues and eigenvectors) to achieve clustering. This method integrates the concept of Coulomb force with KNN-based neighborhood rules, modeling vehicles’ relative motion behaviors (speed, direction, distance) as virtual attractive and repulsive forces. Vehicles moving in the same direction with similar speeds generate positive attractive forces, making them more likely to be grouped into the same cluster. Meanwhile, the KNN constraint limits neighbor relationships to avoid excessive cluster expansion and to maintain structural stability. However, in large-scale and highly dynamic vehicular networks, continuous eigenvalue and eigenvector decomposition of the Laplacian matrix involves O(N^3^) computational complexity, making real-time execution difficult.

Furthermore, numerical instability may arise due to sparsity or poor matrix conditioning, and when eigenvalues are too close or multiple, it becomes challenging to determine the number of clusters accurately. In addition, the number of eigenvectors (k) must be pre-specified, which can lead to unstable results if set improperly. Frequency reconstruction and decomposition are required as the topology changes rapidly, reducing efficiency and stability.

In [29], some studies have also proposed a clustering method based on GNN (Graph Neural Network), which simultaneously considers the graph structure among nodes and multi-dimensional feature information (such as speed, position, acceleration, and vehicle dimensions) to identify behaviorally similar vehicles and form clusters. This approach constructs a global static vehicle graph at each time snapshot. It uses the graphSAGE (SAGEConv) model within the GNN to aggregate features from neighbors within two hops, generating vector embeddings for each node. Finally, these node embeddings are fed into the K-means algorithm for clustering, mapping the learned behavioral similarities into actual cluster formations. In the case of same-direction highways and a single vehicle type, the method based on GNN and K-means improves stability and coverage. Nevertheless, incorporating static vehicle dimensions in multi-vehicle-type or urban scenarios may bias the clustering results toward vehicle-type similarity rather than actual behavioral similarity. At the same time, the hidden-layer learning process of GNN further reduces interpretability, making it difficult to explain why certain vehicles are grouped.

In [26], the elbow method combines Gaussian similarity with vehicle speed and position to calculate motion similarity between vehicles. The elbow method is first used to dynamically determine the optimal K value, addressing the common issue in K-means where the K value is challenging to set and often leads to poor cluster convergence. Once the K value is determined, nodes are clustered based on motion similarity to enhance the stability and accuracy of the clustering. The algorithm calculates a weight for cluster head selection based on node degree (i.e., the number of adjacent nodes that can directly communicate with the candidate node) and average link duration. This helps reduce end-to-end delay and improves node throughput. However, despite these improvements, several limitations remain. The initial cluster centers in K-means are still randomly selected, leaving their inherent sensitivity to initialization unresolved and potentially causing unstable clustering results. The motion similarity metric only considers speed and position through a Gaussian-based index, which may not fully capture real vehicular behavior since other critical factors, such as acceleration or directional variation, are excluded. In addition, the cluster head selection process introduces extra computational and communication overhead, as it requires continuous calculation of motion similarity, node degree, and average link duration ratio.

In addition, there is a study that combined K-means and DBSCAN in [30], selecting the algorithm based on different requirements: when vehicle distribution is uneven and it is necessary to filter out low-density or safety-zone vehicles, DBSCAN is used to detect high-density vehicle groups, and the cluster head is chosen as the node closest to the RSU to implement a far-field communication center technique, thereby reducing channel load. Conversely, when there is a need to form efficient near-field communication clusters, K-means is applied to establish centroid structures based on the relative distances between vehicles, selecting the vehicle closest to the cluster centroid as the cluster head so that cluster members are concentrated around the center to support high-frequency real-time message exchange. However, using two different CH selection criteria—RSU proximity in DBSCAN and centroid proximity in K-means—introduces a fundamental conflict that can lead to frequent CH re-selection and unstable clusters under high vehicle mobility. Since the study does not provide a direct evaluation of cluster lifetime, CH switching frequency, or re-clustering overhead, the actual stability of the clustering process remains uncertain, making this the method’s most critical limitation.

In [27], as for DBSCAN-based techniques, LEADER is an improved DBSCAN method (CDS-DBSCAN). This approach applies a sliding time window Δ*t*, where Δt denotes a fixed-length continuous observation interval that captures node mobility patterns over time rather than relying on instantaneous positions. Within each window, it forms clusters based on the inter-node distance threshold (ε) and the minimum number of points (minPts ≥ 4), where ε means the maximum spatial radius within which two nodes are considered neighbors, and the minimum number of points minPts, where minPts represents the density requirement by specifying the minimum number of neighbors needed to qualify a region as a cluster core. It then selects representative cluster heads using group centrality metrics (such as betweenness and closeness) combined with neighbor count. In [31], another method is a resource-aware DBSCAN re-clustering technique, which dynamically adjusts DBSCAN parameters based on RSU resource allocation. This approach reassigns nodes from overloaded RSU clusters to less-loaded RSUs, resulting in a more balanced distribution of vehicular nodes within clusters and ultimately achieving RSU load balancing. However, both methods reveal critical clustering limitations. These global or semi-global centrality computations in the LEADER algorithm impose significant computational and communication overhead in highly dynamic VANETs, and high-centrality nodes may still be short-lived due to mobility, leading to frequent leader changes. In contrast, the resource-aware DBSCAN approach relies solely on positional proximity (ε tied to DSRC coverage radius) and RSU signal strength for cluster association, which lacks an endogenous cluster head selection mechanism. As a result, cluster heads may be positioned at the cluster boundary rather than centrally, leading to inefficient intra-cluster communication and frequent oscillations when clusters span multiple RSUs. The authors themselves acknowledge that cluster stability and handover overhead remain unresolved.

In [32], one clustering method integrates the GWO (Grey Wolf Optimizer) with the AWCA (Adaptive Weighted Clustering Algorithm) regarding bio-inspired optimization approaches. In this approach, GWO searches for the optimal combination of weights corresponding to four vehicle node features (residual energy, mobility, node degree, and communication cost), enabling the scoring mechanism to adjust node role evaluation criteria according to environmental changes dynamically. Each grey wolf represents a candidate weight vector, and a combination of energy consumption, cluster stability, and communication overhead determines its fitness. Within the optimizer, the α wolf denotes the best current solution and leads the search direction, the β wolf represents the second-best solution providing supporting reference, and the δ wolf represents the third-best solution that contributes to exploration diversity, while the remaining wolves (Ω) represent other candidate solutions that adjust their positions according to the α, β, and δ wolves. The final converged weights are then applied within AWCA to select cluster heads and establish clusters. Although the proposed GWO-AWCA framework improves adaptability by dynamically optimizing clustering weights, several issues may undermine its performance in highly dynamic VANET scenarios. Since GWO relies on AWCA’s input features (residual energy, node degree, load, mobility), rapid topology changes can cause these features to fluctuate significantly, disrupting the optimizer before convergence and resulting in unstable or inconsistent weight assignments. Moreover, GWO is an iterative metaheuristic that requires multiple fitness evaluations to update the α, β, and δ wolves; if the network topology evolves faster than the optimizer’s convergence rate, the computed weights may already be outdated, leading to ineffective cluster head selection. In extreme cases where instability dominates, the system may exhibit the practical absence of a well-formed cluster.

To sum up, in [26], the motion similarity between vehicles must be calculated. In [27], a fog computing layer and leader election were used. In [28], vehicle mobility is modelled by using the Coulomb law. Furthermore, some clustering methods by machine learning or neural networks need several iterations of computation [29,30]. In our proposed scheme, vehicles only need to know the number of the neighbors nearby. Moreover, in [31], the resource availability of base stations must be computed. In [32], the authors proposed a metaheuristic-based clustering framework based on real-time metrics such as residual energy, traffic density, and communication overhead computed in advance. Most elaborate approaches are comprehensive with complexity, as mentioned in the literature. Here, in the proposed mechanism, we focus on the algorithm that is feasible and easy to be realized.

In addition, to carry on our previous research [3,4], we found that organizing vehicles into groups can enhance the safety of the vehicle networks with LiDAR assistance. However, the success rate and reliability of grouping vehicles is an important issue. Also, enhancing existing V2V communication mechanisms remains a key factor in ensuring that emergency messages can be transmitted both timely and accurately. To address this, a study proposed an innovative strategy that integrates prime number-based lane encoding, hop count tagging, and message priority control to tackle existing issues such as excessive broadcast redundancy, high message delay, and difficulty in filtering out irrelevant warnings. In this approach, each lane is assigned a unique prime number, and the product of the prime numbers of accident-related lanes is embedded into the broadcast message. This allows receiving vehicles to quickly determine whether the warning is relevant to them through a simple divisibility check. Additionally, hop count tags are used to assess the distance and severity of the event, while the priority control mechanism ensures that emergency messages are transmitted with minimal interference. Furthermore, the study incorporates GPS positioning technology to accurately determine the vehicle’s lane location during startup or lane changes, aligning it with the corresponding prime number encoding. This enables the system to automatically identify spatial position and contextual relevance even in complex traffic scenarios (e.g., multilane roads or intersections), significantly improving the targeting and spatial filtering of warning messages while reducing GPS usage frequency to save communication and computational resources. Simulation results show that the proposed mechanism effectively reduces communication load and warning delay, thereby improving the efficiency and accuracy of message transmission as well as drivers’ reaction time [3,4].

In order to strengthen the connectivity of vehicles, based many specifications, we proposed a mechanism to aggregate groups of vehicles to cooperatively communicate with each other. This method fulfills most of the vehicle system requirements and can be used to group vehicles on the road briefly.

## 3. System Architecture

**Figure 4:**
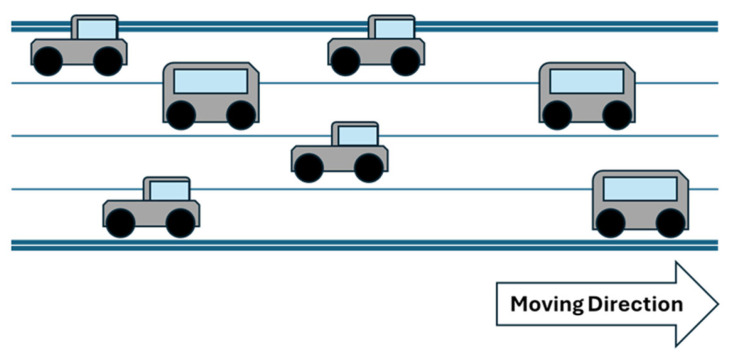
The concept of a one way, four lanes road. This is a schematic diagram; the lines in the middle represent the lane lines.

As shown in Figure 4, a model considered here with the condition of a one-way road with four lanes was proposed where the total length of the road is 250 m, the width of a single lane is 3 m, the distance between the center points of vehicles on the same lane is at least 6 m, the distance between the center points of vehicles when running parallel to each other is 3 m, and the radius of the vehicle communication range is within 10 m. Under these conditions, we assume that the road length is fixed. The experiments are performed by adjusting the total number of vehicles. In the network environment, it is assumed that the greater the number of routers and nodes is, the larger the scale of the network coverage is. They increase proportionally. In addition, it is also worth noting that many roadside routers lead to an increase in construction costs. However, low-density-router deployment is expected to result in limited coverage of communication services.

In addition, within the context of the global network topology, to accomplish the task of data exchange, there must at least be regular nodes, routers, gateways, and cloud devices. Therefore, when allocating a cluster of vehicles, one vehicle will serve as the cluster head acting as the router. After setting the simulation environment conditions, the conditions are input into the program, and vehicle positions are randomly generated within the defined range according to the set conditions. Once the vehicle positions are deployed, clusters are formed in the order of vehicle numbers. If the distance between vehicles is within a radius of 10 m, they will be grouped into the same cluster.

Since the roles of nodes in the cluster include at least one router and three general communication nodes, the number of nodes in the cluster should be greater than three. In the case of randomly deploying nodes, it is possible that the distribution of nodes in certain areas may be dense or sparse. To determine a reasonable clustering method, the K-means algorithm is the first clustering method used for evaluation. Taking the two-dimensional node distribution diagram as an example, the clustering process can be divided into the following steps:

Step 1: If the total number of nodes is not large, after manually interpreting the visualization chart, the expected number of clusters can be set to 3, and the K value should be set to 3.

Step 2: The algorithm generates K reference points randomly based on the given K value to perform the clustering task.

Step 3: After completing the reference point configuration, all nodes are traversed, and the Euclidean distance formula is used to calculate the distance between each node and the reference points for clustering. For example, if node 1 is closest to reference point 1 after calculating the distances to all three reference points, it is assigned to cluster 1.

Step 4: Keep adjusting the positions of the reference points. This time, the reference points are not randomly assigned but calculated by summing the data within each cluster and dividing by the number of data points in the cluster.

Step 5: Repeat step 4 until the positions of the reference points no longer change.

After going through the steps as mentioned above, we can observe that this clustering method is not suitable for the scenario in the study. When the number of nodes reaches hundreds or even thousands, the K value can no longer be determined manually. Therefore, a more ideal method is needed to determine the K value. In the K-means algorithm, there is a clustering method called the “elbow method”, and the related steps are described as follows:

Step 1: Set the upper and lower limits for the K value. For example, if the distribution of nodes suggests that the expected K value might be around 4, then the range for K should be between 1 and 10.

Step 2: The algorithm generates K reference points randomly based on the given K value to perform the clustering task.

Step 3: After completing the reference point configuration, all nodes are traversed, and the Euclidean distance formula is used to calculate the distance between each node and the reference points for clustering. For example, if node 1 is closest to reference point 1 after calculating the distances to all three reference points, it is assigned to cluster 1.

Step 4: Keep adjusting the positions of the reference points. This time, the reference points are not randomly assigned but calculated by summing the data within each cluster and dividing by the number of data points in the cluster.

Step 5: Repeat step 4 until the positions of the reference points no longer change.

Step 6: After completing the above steps, calculate the distance from each data point in each cluster to the cluster centroid. This distance is the SSE.

Step 7: By repeating the above steps, you can obtain the SSE corresponding to each K value. Then, from the relationship between the K values and SSE, identify the point where the decrease in SSE becomes relatively flat, which is the “elbow point”, this elbow point indicates the optimal number of clusters. The pseudocodes are listed in Table 2 and Table 3.

However, the elbow method still cannot be considered an ideal clustering method. Because we need to set the communication range for individual nodes, we impose a minimum distance constraint between two nodes along the *Y*-axis, and enforce a minimum number of nodes per cluster for our data. This means that the density of the allocated nodes will affect cluster assignment. Therefore, the DBSCAN (Density-Based Spatial Clustering of Applications with Noise) algorithm would be a more ideal clustering method. DBSCAN has three core concepts: the core point, border point, and noise point. Before clustering, two important parameters should be set: ε and minimum points, the algorithm clusters based on these two conditions. For example, if there are five points in a region, and expanding from point p1 as the center with a radius of ε, four points can be found, and expanding from point p2 as the center, only two points (including p1) are found, so p1 is the core point and p2 is the border point. A noise point refers to a node that exists independently in the data and cannot be assigned to any cluster.

**Figure 5:**
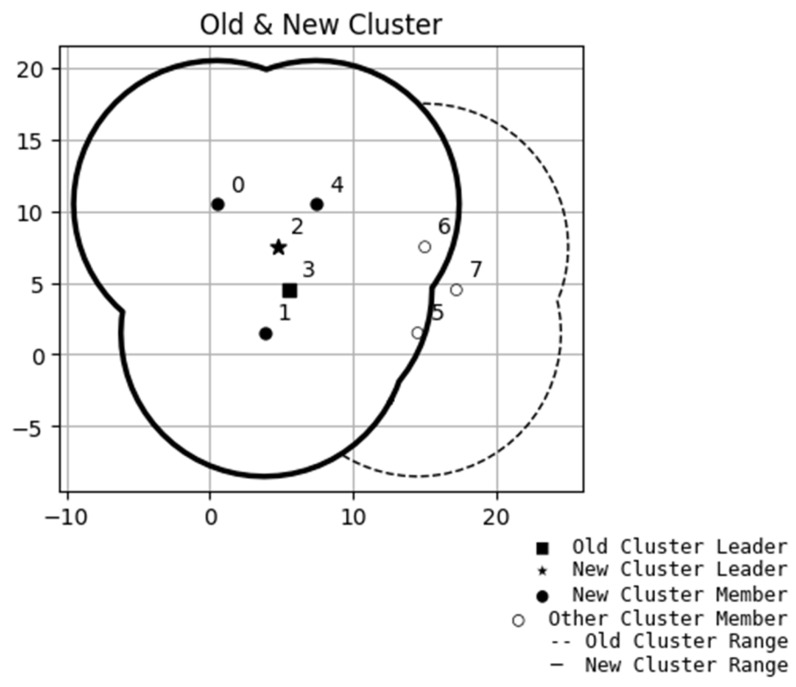
Proposed algorithm of nodes allocated to a cluster. The numbers listed are the serial numbers of the nodes (cars).

In the study, after randomly deploying the node positions, the algorithm begins traversing from either the node closest to the origin of the *x*-axis or the one farthest from it. During the traversal, neighboring nodes are identified based on the communication radius ε, along with the condition that each cluster must contain a minimum number of nodes. After completing the traversal of a node, the total number of its neighbors and their respective IDs are recorded. The same procedure is then applied to the next node. This process iterates until all nodes have been traversed. Once the initial traversal is complete, the algorithm officially begins the clustering process. In this work, the number of neighboring nodes can be used as a clustering criterion. For example, clustering can start from nodes with the most neighbors or from those with the fewest. Moreover, nodes that have already been marked as neighbors in a cluster cannot be assigned to another cluster, even if they also have neighbor relationships with other nodes. As illustrated in Figure 5, following the completion of node distribution, Node 3 initially has neighboring nodes 0, 1, 2, 4, 5, and 6. During the actual clustering process, Node 0 is the first to satisfy the clustering criteria and is selected to initiate the clustering procedure. Given that several of Node 0’s neighbors overlap with those of Node 3, Nodes 0 through 3 are consequently grouped into the same cluster. As a result, Node 3’s original neighboring nodes, specifically Nodes 5 and 6, are excluded from this cluster and are thus not assigned to the same group as Node 3. Therefore, any node that has already been assigned to a cluster will be removed from the neighbor lists of other nodes. Additionally, it is worth noting that to ensure continuous communication within a network topology, the presence of a backup router helps with this requirement. This way, if the primary router fails, a backup router can promptly take over, maintaining communication among the cluster nodes and enhancing the reliability of traffic systems.

Therefore, according to the clustering method as above description, the experiment is conducted based on the following conditions, and a relatively reasonable number of clusters will be found as a reference from the experiment result:

- The road length is 250 m.
- The road has 4 lanes in total.
- The road width is 3 m.
- The shortest distance between node centers in the same *Y*-axis direction is 6 m.
- The communication radius is 10 m.
- The minimum number of nodes required for the cluster should be larger than 4.
- The minimum number of nodes in the road is 3 and the maximum is 250.
- The number of tests for each group of nodes is 1000 times.

In our opinion, the ideal scenario is one in which every vehicle on the road can be assigned to a cluster. However, this is not always achievable in the real world. Therefore, we aim to identify the set of nodes for which the clustering assignment rate is no less than 90%. During this process, if the previous set meets the 90% threshold but the next set does not, the search must continue until a set is found where the assignment rate is at least 90%, and all subsequent sets also maintain an assignment rate of 90% or higher.

**Figure 6:**
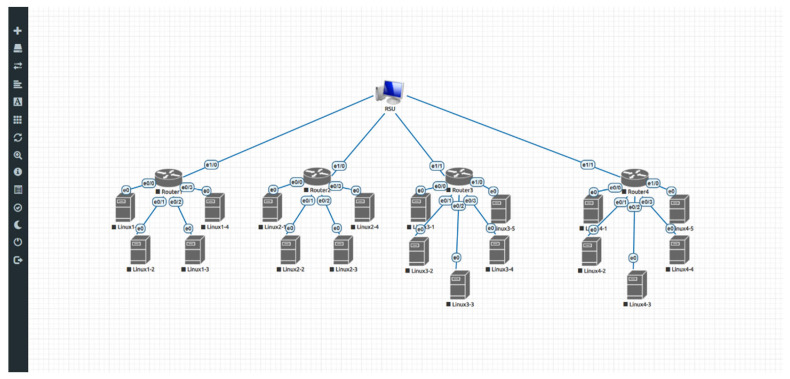
Network topology simulation environment established in EVE-NG. Arrows and symbols are automatically generated by the development tool to represent nodes and interface labels.

After completing the exploration for the optimal number of cluster nodes, we can consider using the EVE-NG network emulator to construct a partial simulation environment based on the generated network topology. For example, in Figure 6, there are four clusters shown in the diagram. Each cluster consists of approximately 4 to 5 regular nodes and one router node. To enable communication between all nodes in a cluster and the cloud, each regular node first connects to the router, which then connects to a host acting as the RSU. Once the network topology is configured, we can use iperf to perform traffic testing in order to evaluate the average data throughput available to each node.

To perform random node distribution and cluster assignment, we developed a program using the C programming language. The implementation includes three primary functions, which are responsible for random node placement, cluster allocation, and the assignment of routers and backup routers within each cluster.

In the process of random node placement, node positions are assigned based on several parameters, including the total number of nodes, road length, number of lanes, lane width, and the minimum inter-vehicle distance. If a newly generated position overlaps with an existing node or the distance to a neighboring node is less than the defined minimum, a new random position is generated for that node. If repeated attempts fail, indicating that there is insufficient space to place additional nodes, the program terminates.

At the next stage of the cluster allocation process, the cluster assignment process begins by traversing all nodes to calculate the number of neighbors and record their IDs. For each traversed node which has not been assigned to any cluster, the calculation of the Euclidean distance between the traversed node and the neighbor is performed. If the distance is within a predefined threshold ε, the node is marked as a neighbor. After the neighbor discovery process is completed for a node, a neighbor list is constructed based on the total number of identified neighbors.

Once all nodes have been traversed, the cluster allocation begins. A node is allocated to the prior cluster based on having either the minimum or maximum number of neighbors, provided that it has at least three neighbors. After a new cluster is allocated, all nodes are traversed again to remove the node which has been assigned to a new cluster from the pool of unassigned nodes. This procedure repeats iteratively until no further cluster assignments can be made.

After clustering is completed, the next step involves selecting appropriate routers and backup routers for each cluster. Initially, candidate nodes are identified based on whether their distances to other nodes in the cluster are not greater than 10. At this point, a question may arise as to why some nodes within a cluster may have distances greater than 10 between them. This is because the clustering process is based on a single node’s perspective when discovering neighbors, and thus does not guarantee the distance is not less than or equal to 10 between each node within a cluster. This highlights the importance of the cluster head serving as the router.

Once the candidate nodes are identified, each of them undergoes an additional round of Euclidean distance calculations with all other nodes in the same cluster. The average distance is then computed for each candidate, and the node with the smallest average distance is selected as the cluster head (primary router). The remaining candidates are designated as backup routers; the backup priority is determined based on the average distance between each backup node and the nodes within the cluster, sorted in ascending order. This strategy helps minimize overall communication distance among nodes, and ensures that in the event of a primary router failure, a backup router can promptly take over routing responsibilities. As a result, communication within the cluster can be maintained, thereby enhancing the overall reliability of the traffic system.

## 4. Experiment Results

**Figure 7:**
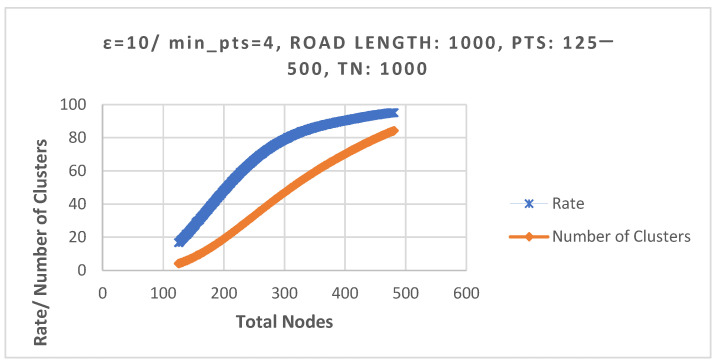
Comparison of success rate and average number of clusters. It indicates that as the number of nodes increases, the number of clusters also increases.

In the experimental section, we first explored the clustering success rate and the average number of clusters under a fixed road length condition. In Figure 7, the road length was set to 1000 m, and the total number of nodes ranged from 125 to 500. The results showed that when the total number of nodes reached 394, a 90% clustering success rate could be achieved, with an average of 69 clusters formed. However, when the total number of nodes increased to 481, the clustering success rate reached the highest peak. From that point onward, due to environmental limitations preventing the accommodation of more nodes, the failure rate of node deployment increases, which in turn leads to a decrease in clustering success rate. The parameters are listed in Table 4.

**Figure 8:**
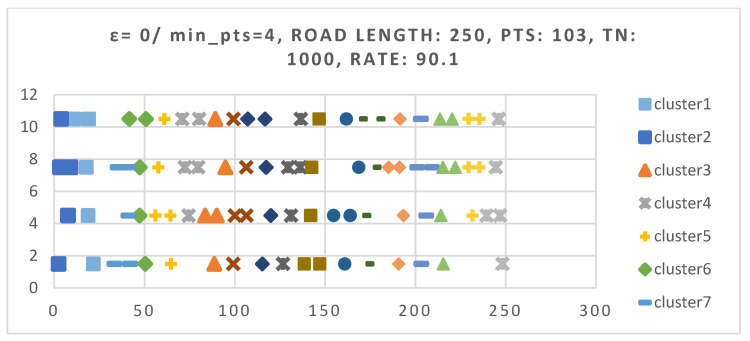
The result of self-organized vehicle nodes and distribution of clusters. The symbols and colors denoted different clusters.

In order to examine the relationships between nodes and the rationality of cluster distribution, we also adjusted the simulated road length back to 250 m as shown in Figure 8. Due to the shortened road length, the total number of nodes was reduced to 250. According to the experimental results, when the total number of nodes was 103, the clustering success rate reached 90%, with an average of 18 clusters formed. Therefore, we selected a dataset from the experimental results that contains 18 clusters to generate a visual representation of the node distribution. It can be observed that most of the cluster head nodes are located near the central line, which satisfies the requirement for routers to maintain the shortest communication distance with their cluster members.

In addition, we conducted experiments focusing on four clustering directions. Firstly, the so-called quadrant-based clustering directions refer to clustering the nodes in the environment from the bottom-left to the top-right, from the top-left to the bottom-right, from the bottom-right to the top-left, and from the top-right to the bottom-left. The purpose of the experiment is to determine whether the direction of clustering affects the clustering success rate and results when the total number of nodes remains the same and when the number of cluster members varies. The terms “more” or “fewer” cluster members refer to whether the clustering process begins with nodes that have more neighbors or with those that have fewer neighbors. By applying these different clustering strategies, we aim to evaluate the clustering success rate and the average number of clusters formed, in order to analyze which strategy yields better performance.

Therefore, in this experiment, two cases were designed based on the number of cluster members—one with fewer and another with more. For each case, four scenario-based experiments were developed according to four clustering directions. As the experimental results in Table 5 and Table 6 show, it can be observed that regardless of whether clustering is performed under the condition of fewer or more cluster members, the differences in clustering success rate and average number of clusters across the four quadrant directions are not obvious. Therefore, it can be concluded that in practical clustering tasks, using just one clustering direction is sufficient.

Moreover, from the perspective of cluster member count, under the same road length and total number of nodes, clustering under the condition of fewer cluster members consistently achieves a higher clustering success rate, and the average number of clusters formed is also higher than that under the condition of more cluster members. Thus, whether the clustering is performed in a suburban or urban traffic environment, it is recommended to adopt the strategy of clustering with fewer members. This not only ensures a higher success rate but also results in a greater number of clusters, which in turn reduces the load on individual cluster heads and helps achieve load balancing, thereby improving communication quality.

**Figure 9:**
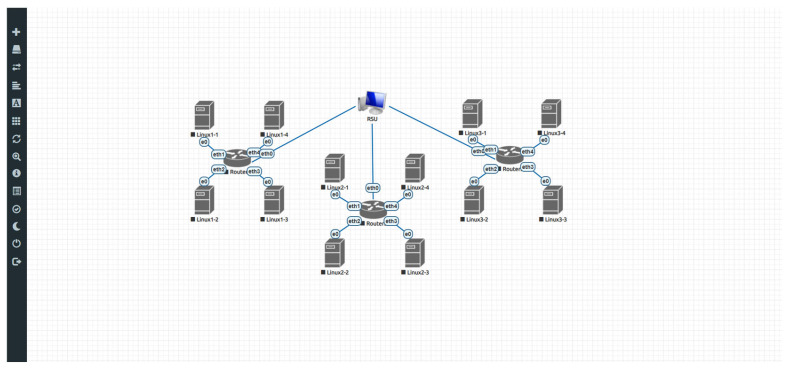
An example topology established in EVE-NG for the experiment. Arrows and symbols are automatically generated by the development tool to represent nodes and interface labels.

Based on the aforementioned clustering method that we proposed, data transmission rate experiments were subsequently conducted. As shown in Figure 9, a network topology consisted of one server (in the cloud) and three clusters, with each cluster comprising a cluster head and a total of five nodes in this design. The experiment was conducted using the EVE-NG platform, where the server refers to the EVE-NG host itself. The cluster heads of the three clusters were implemented using VyOS, which provides routing capabilities within the architecture. Each VyOS instance was allocated 2 CPUs and 2 GB of RAM. The remaining nodes were built using Ubuntu Desktop, with each assigned 2 CPUs and 1 GB of RAM.

In this architecture, regular nodes are equipped with DHCP client functionality, allowing them to obtain an IP address from the router. Therefore, VyOS serves both as a router and as a DHCP server/client. In addition to acquiring an IP address from the server, it also provides IP address assignments to regular nodes for subnetwork. Taking Cluster 1 as an example, the cluster head can fulfill these requirements through the following configuration:

- Starting the configuration
  Configure

- DHCP get IP from EVE-NG
  set interfaces ethernet eth0 address dhcp

- Set Bridge
  set interfaces bridge br0 address 192.168.1.1/24
  set interfaces bridge br0 description ‘LAN-Bridge’
  set interfaces bridge br0 member interface eth1
  set interfaces bridge br0 member interface eth2
  set interfaces bridge br0 member interface eth3
  set interfaces bridge br0 member interface eth4

- DHCP Server for Linux Nodes
  set service dhcp-server shared-network-name LAN subnet 192.168.1.0/24 subnet-id 1
  set service dhcp-server shared-network-name LAN subnet 192.168.1.0/24 option default-router 192.168.1.1
  set service dhcp-server shared-network-name LAN subnet 192.168.1.0/24 lease 86400
  set service dhcp-server shared-network-name LAN subnet 192.168.1.0/24 range start 192.168.1.2
  set service dhcp-server shared-network-name LAN subnet 192.168.1.0/24 range 0 stop 192.168.1.200

- OSPF for auto route
  set protocols ospf area 0 network 192.168.139.0/24
  set protocols ospf area 0 network 192.168.1.0/24
  Updating the configuration
  commit
  save
  exit

- For all nodes
  ssh-keygen -t rsa
  ssh-keyscan -H 192.168.139.129 >> ~/.ssh/known_hosts
  ssh-copy-id -i ~/.ssh/id_rsa.pub root@192.168.139.129First bullet;
  Second bullet;
  Third bullet.

After completing the network configuration, a priority-based resource allocation method was proposed to simulate the behavior of nodes transmitting data within the network. In this method, priorities are classified into three levels: high, medium, and low, corresponding to the values 3, 2, and 1, respectively. The process begins with assigning priorities to clusters. The priority of a cluster is determined based on the total number of nodes it contains: the more nodes in a cluster, the higher its priority. In the proposed example, each cluster contains exactly five nodes, so all clusters are assigned equal priority. Next, priority allocation is applied to the nodes within each cluster. It is important to note that the cluster head serves as the data gateway for all nodes, including itself; therefore, it must be assigned a high priority, and then assign priorities to the remaining nodes and perform weighted calculations. In addition to priority assignment, a Poisson distribution is used to estimate the transmission time for each node. For example, suppose the five nodes in a cluster including the cluster head and the related priorities are assigned “High”, “Medium”, “High”, “Low”, and “Low”, sequentially.

To examine the effectiveness of the proposed method for data packets transmitted, two schemes are designed for comparison. The first one is the burst transmission test, in which all nodes attempt to send data to the server at almost the same time, denoted Case I. The other one is a backoff delay transmission test, where each node transmits data to the server sequentially with delay time exponentially distributed, named Case II. The expected backoff delay time for each node in the simulation program is calculated using the following exponential distribution equation:

T_f_ = −log(1.0 − (rand()/(RAND_MAX + 1.0)))/λ_node_

where λ_node_ means the arrival rate of transmitted data bits. The results are shown in Table 7 and Table 8 as below.

For information security, in the experiments, all nodes transmit data to the server using “scp”, which is a data transfer method based on SSH. This method employs asymmetric encryption and depends on the SSH public key authentication mechanism to complete the data transmission process. In Table 7 and Table 8, kBps denotes kilobytes (packets) being sent, MBps means megabytes (packets) being sent, and Mbps (with a lower-case “b”) denotes megabits per second being transmitted. The experiment results show that vehicle nodes need to wait a delay time before transmission. Case II achieves good performance in avoiding collisions, especially in congested urban regions.

## 5. Conclusions

In this paper, a mechanism to group vehicles into several clusters is proposed. With the benefit of vehicle connectivity, vehicles are communicate with each other easily and cooperatively to improve traffic safety. The K-means algorithm is a way to solve this problem, but the K value is difficult to determine. In the proposed algorithm, vehicles only need to discover the neighboring cars by using light detection and ranging (LiDAR) sensors. The limitation of the proposed method here is that some free vehicles (nodes) could not be assigned to a cluster because of far distance or deep fading of signals. However, the numerical results revealed that a success rate of over 90% can be achieved. To summarize the contributions of this paper, the advantages of the proposed mechanism are two-fold. First, with the experimental platform EVE-NG, the results showed that a clustering success rate of over 90% could be achieved. Secondly, furthermore, the experiment results reveal that data packets queued in each car to wait a random time, exponentially distributed, before transmission outperform data packets sent in a moment, without backoff waiting, in a short period of time. As shown in Table 7 and Table 8, the total throughput in Case II is more than four times that of Case I. In the future, we will try to organize vehicles using AI tools if most auto-driving cars are popular and already equipped with high performance on-board units, (OBUs) on level four.

## Figures and Tables

**Table 1:** Acronyms and abbreviations.

Acronyms and Abbreviations	
3GPP	Third Generation Partnership Project
AODV	Ad Hoc On-Demand Distance Vector Routing
BSM	Basic Safety Messages
BSS	Basic Service Set
C-ITS	Cooperative Intelligent Transport Systems
C-V2X	Cellular Vehicle-to-Everything
C2C-CC	Car-to-Car Communication Consortium
CAGR	Compound Annual Growth Rate
CAV	Connected and Autonomous Vehicle
CCAM	Cooperative Connectivity and Automated Mobility
CCH	Control Channel
CEN	Comité Européen de Normalisation
CoAP	Constrained Application Protocol
CPU	Central Processing Unit
CRL	Certificate Revocation List
D2D	Device-to-Device
DSR	Dynamic Source Routing
DSRC	Dedicated Short-Range Communication
DT	Digital Twin
DYSCH	Dynamic Scheduling
ECC	Elliptic Curve Cryptography
ECDSA	Elliptic Curve Digital Signature Algorithm
ECIES	Elliptic Curve Integrated Encryption Scheme
EDCA	Enhanced Distributed Channel Access
EPD	EtherType Protocol Discrimination
ETSI	European Telecommunications Standards Institute
EVE-NG	Emulated Virtual Environment Next Generation
HetVNET	Heterogeneous Vehicular Networks
ITS	Intelligent Transportation System
LiDAR	Light Detection and Ranging
LTE-V2X	Long Term Evolution-Vehicle-to-Everything
OBU	On-Board Unit
PLME	Protocol Layer Management Entity
QUIC	Quick UDP Internet Connections
RREQ-RREP	Route Request-Route Reply
SPDU	Security Services Protocol Data Unit
UTC	Coordinated Universal Time
VANET	Vehicular Ad Hoc Network
WAVE	Wireless Access in Vehicular Environments
XGBoost	Extreme Gradient Boosting

**Table 2:** Functions of assignment of vehicles on a road.

**Pseudocode:** GenerateVehiclePositions(point_count, points)
**1:** possible_y ← [LANE_WIDTH/2, **2:** LANE_WIDTH/2 + LANE_WIDTH, **3:** LANE_WIDTH/2 + 2 × LANE_WIDTH, **4:** LANE_WIDTH/2 + 3 × LANE_WIDTH] //Lane center positions **5:** Print horizontal separator **6:** for i ← 0 to point_count − 1 do **7:** valid_position ← FALSE **8:** tries ← 0 **9:** while valid_position = FALSE and tries < MAX_TRIES do **10:** p.x ← random value in [0, ROAD_LENGTH) **11:** p.y ← randomly select from possible_y **12:** valid_position ← TRUE **13:** if i ≠ 0 then **14:** for j ← 0 to i − 1 do **15:** if is_too_close(p, points[j]) then **16:** valid_position ← FALSE **17:** tries ← tries + 1 **18:** break **19:** end if **20:** end for **21:** end if **22:** end while **23:** if tries = MAX_TRIES then **24:** Print “Failed to find valid position for vehicle”, i **25:** return FALSE **26:** end if **27:** points[i] ← p **28:** points[i].cluster_id ← 0 **29:** Print “NO.”, i, “ (x, y): “, p.x, p.y **30:** end for **31:** Print newline **32:** return TRUE
**Pseudocode:** Clustering with Varying Vehicle Counts
**1:** for num_pts ← min_pts to max_pts do **2:** for tries ← 0 to max_tries − 1 do **3:** points ← allocate array of size num_pts **4:** initialize points with zeros **5:** **6:** if generate_vehicle_positions(num_pts, points) = FALSE then **7:** free points **8:** continue **9:** end if **10:** **11:** sort points by location using qsort **12:** Print “List new location info after sorting:” **13:** for idx ← 0 to num_pts − 1 do **14:** Print “NO.”, idx, “(x, y):”, points[idx].x, points[idx].y **15:** end for **16:** Print newline **17:** **18:** checkRet ← ClusterAssign(points, num_pts, ε, MIN_NEIGHBORS) **19:** if checkRet = TRUE then **20:** if print_results(points, num_pts) = FALSE then **21:** free points **22:** continue **23:** end if **24:** **25:** total_conn ← total_conn + (conn_pts/num_pts) × 100 **26:** FindRouterAndBackups(points, num_pts, total_clusters) **27:** conn_pts ← 0 **28:** else **29:** Print “checkRet = FALSE” **30:** end if **31:** **32:** free points **33:** end for **34:** end for
**Algorithm:** ClusterAssign(points, point_count, ε, minPts)
**Input:** points: a list of points to be clustered point_count: number of points ε: distance threshold (epsilon) minPts: minimum number of neighbors required to form a cluster **Output:** Clusters assigned via cluster_id for each point **Steps:** Initialize cluster_id ← 1 Allocate check_points[point_count] ← 0 Repeat For each unclustered point i, do: Reset has_neighbor ← 0 Initialize check_points ← 0 For each point j ≠ i and unclustered: If distance(points[i], points[j]) ≤ ε then Mark check_points[j] ← 1, increment has_neighbor Assign points[i].has_neighbor ← has_neighbor Allocate and initialize points[i].neighborList of size has_neighbor Copy marked neighbor indices into points[i].neighborList End For Find the point with minimum has_neighbor ≥ minPts, call it hasMinNb If no such point exists, free memory and break Assign cluster_id to points[hasMinNb] and all its neighbors For all other points i ≠ hasMinNb: Remove any assigned neighbors from points[i].neighborList If neighbors removed: Recompute has_neighbor, update neighborList Increment cluster_id Free all neighborList for all points Until no more valid clustering possible Free check_points Return TRUE

**Table 3:** Functions of assignment of cluster heads.

**Algorithm:** function FindRouterAndBackups(data, num_points, num_clusters)
**Input:** data: Array of Points with cluster assignments num_points: Total number of points num_clusters: Total number of clusters **Output:** Best router and backup nodes for each cluster **Steps:** For each cluster_id from 1 to num_clusters do: Initialize count ← 0 For i from 0 to num_points − 1 do: If data[i].cluster_id = cluster_id then count ← count + 1 End For If count = 0 then Return FAILURE Allocate chPoints of size count ptIdx ← 0 For i from 0 to num_points − 1 do: If data[i].cluster_id = cluster_id then chPoints[ptIdx] ← data[i] ptIdx ← ptIdx + 1 End For Initialize goodNodes [0..count−1] ← 1 For i from 0 to count − 1 do: For j from 0 to count − 1 do: If i ≠ j and EuclideanDistance(chPoints[i], chPoints[j]) > 10.0 then goodNodes[i] ← 0 Break End For Initialize validNodes as empty list For i from 0 to count − 1 do: If goodNodes[i] = 1 then sumDist ← 0 For j from 0 to count − 1 do: If i ≠ j then sumDist ← sumDist + EuclideanDistance(chPoints[i], chPoints[j]) End For avgDist ← sumDist/(count − 1) Append (i, avgDist) to validNodes End For If validNodes is empty then Continue to next cluster Sort validNodes by avgDist in ascending order Assign first node in validNodes as router Remaining nodes are backup routers in sorted order End For Return SUCCESS

**Table 4:** Experiment parameters.

Parameters	Description
dot11OCBEnabled	Outside the Context of a BSS, no BSS mode while it is true.
Ts0Duration	The duration of time slot 0, for CCH.
Ts1Duration	The duration of time slot 1, for SCH.
ε	Communication Radius
λ_cluster_	Data transmission frequency of the cluster
λ_node_	Data transmission frequency of the node
T_f_	Estimated transmission time

**Table 5:** The nodes with fewer neighbors are allocated to a cluster first.

Scenario	Total Nodes	Success Rate (%)	No. of Clusters
1	120	97.50	21
2	120	97.50	21
3	120	94.20	20
4	120	94.20	20

**Table 6:** The nodes with more neighbors are given prior consideration over those with fewer neighbors.

Scenario	Total Nodes	Success Rate (%)	No. of Clusters
1	120	90.00	12
2	120	90.00	12
3	120	91.70	12
4	120	91.70	12

**Table 7:** Case I: nodes send data bits immediately as packets arrive, without waiting.

Burst Transmission											
Cluster1				Cluster2				Cluster3			
	KBps	MBps	Mbps		KBps	MBps	Mbps		KBps	MBps	Mbps
Node1	9909.2	9.7	77.4	Node1	11162.3	10.9	87.2	Node1	12198.6	11.9	95.3
Node2	1969.7	1.9	15.4	Node2	1808.0	1.8	14.1	Node2	1797.9	1.8	14.0
Node3	2116.5	2.1	16.5	Node3	2002.0	2.0	15.6	Node3	1936.9	1.9	15.1
Node4	1940.3	1.9	15.2	Node4	1895.7	1.9	14.8	Node4	1807.6	1.8	14.1
Node5	2140.5	2.1	16.7	Node5	2011.8	2.0	15.7	Node5	1938.9	1.9	15.1
**Total**	18076.2	17.7	141.2	**Total**	18879.9	18.4	147.5	**Total**	19680.0	19.2	153.8

**Table 8:** Case II: nodes back off a random delay time before sending data bits to the server.

Priority Based with Poisson Transmission											
Cluster1				Cluster2				Cluster3			
	KBps	MBps	Mbps		KBps	MBps	Mbps		KBps	MBps	Mbps
Node1	16834.2	16.4	131.5	Node1	16599.0	16.2	129.7	Node1	17138.2	16.7	133.9
Node2	9713.6	9.5	75.9	Node2	10057.6	9.8	78.6	Node2	10026.7	9.8	78.3
Node3	9662.5	9.4	75.5	Node3	10169.4	9.9	79.4	Node3	10228.7	10.0	79.9
Node4	10152.6	9.9	79.3	Node4	9970.1	9.7	77.9	Node4	9866.3	9.6	77.1
Node5	9978.4	9.7	78.0	Node5	9863.9	9.6	77.1	Node5	9886.7	9.7	77.2
**Total**	56341.4	55.0	440.2	**Total**	56659.9	55.3	442.7	**Total**	57146.7	55.8	446.5

## Data Availability

The original contributions presented in this study are included in the article. Further inquiries can be directed to the corresponding author.

## References

[1] https://www.precedenceresearch.com/automotive-v2x-market.

[2] Global Status Report on Road Safety. https://iris.who.int/bitstream/handle/10665/375016/9789240086517-eng.pdf.

[3] Tsai C.S., Du W.K. An Aggressive Wireless Access Data Strategy for Mobile Net-works. Proceedings of the 2007 IEEE International Conference on Systems, Man and Cybernetics.

[4] Tsai C.-S., Du W.-K. (2008). Enhancement of Delivery of Warning Messages for Mobile Networks. J. Networks.

[5] Lim K., Tuladhar K.M. LIDAR: Lidar Information based Dynamic V2V Authentication for Roadside Infrastructure-less Vehicular Networks. Proceedings of the 2019 16th IEEE Annual Consumer Communications & Networking Conference (CCNC).

[6] Sun P., Sun C., Wang R., Zhao X. (2022). Object Detection Based on Roadside LiDAR for Cooperative Driving Automation: A Review. Sensors.

[7] Liu Y., Sun S., Zhang R. Sensing-Assisted Neighbor Discovery for Vehicular Ad Hoc Networks. Proceedings of the 2023 IEEE Wireless Communications and Networking Conference (WCNC).

[8] https://static.nhtsa.gov/odi/inv/2022/INCR-EA22002-14496.pdf.

[9] https://www.its-jp.org/english/about_e/.

[10] https://itsa.org/the-story-of-our-industry/.

[11] https://www.its-uk.org/about/.

[12] https://techcrunch.com/2019/04/23/waymo-picks-detroit-factory-to-build-self-driving-cars/.

[13] FCC Allocates Spectrum in 5.9 GHz Range for Intelligent Transportation Systems Uses. https://docs.fcc.gov/public/attachments/DOC-177370A1.pdf.

[14] Li P., Wu K., Cheng Y., Parker S.T., Noyce D.A. (2024). How Does C-V2X Perform in Urban Environments? Results From Real-World Experiments on Urban Arterials. IEEE Trans. Intell. Veh..

[15] C-V2X vs. DSRC: Which Technology Is Better for Autonomous Vehicles?. https://www.autonomousvehicleinternational.com/features/c-v2x.html.

[16] Nguyen H., Xu X., Guan Y.L. V2V Communications under the Shadowing of Multiple Big Vehicles: II. System Model. Proceedings of the 2019 IEEE 90th Vehicular Technology Conference (VTC2019-Fall).

[17] IEEE Guide for Wireless Access in Vehicular Environments (WAVE) Architecture: 4.3.1 Trial-Use WAVE Standards—Historical. https://ieeexplore.ieee.org/document/7982731.

[18] C-V2X Drives Intelligent Transportation. https://www.qualcomm.com/content/dam/qcomm-martech/dm-assets/documents/cv2x_white_paper-final.pdf.

[19] https://www.car-2-car.org/about-us.

[20] Cooperative Intelligent Transport Systems (C-ITS) Guidelines on the Usage of Standards: 2.4.3 Further Advocates Active in C-ITS. https://www.itsstandards.eu/app/uploads/sites/14/2020/10/C-ITS-Brochure-2020-FINAL.pdf.

[21] Mitigation Techniques to Avoid Interference Between European CEN Dedicated Short Range Communication (CEN DSRC) Equipment and Intelligent Transport Systems (ITS) Operating in the 5 GHz Frequency Range. https://www.etsi.org/deliver/etsi_ts/102700_102799/102792/01.02.01_60/ts_102792v010201p.pdf.

[22] Volkswagen and NXP Deliver Safety to European Roads with World’s Largest Rollout of Communicating Car Technology. https://www.car-2-car.org/fileadmin/documents/Publications/2019-10-17_V2XVWGolf8_FINAL.pdf.

[23] https://c-its-deployment-group.eu/knowledge-base/glossary/.

[24] https://www.itskrs.its.dot.gov/briefings/executive-briefing/signal-phase-and-timing-spat.

[25] Simulating the Effects of Optimal Mobility. https://www.itsinternational.com/its5/its6/its7/its8/feature/simulating-effects-optimal-mobility.

[26] Guo M., Liu Y. A Clustering Method Based on K-Means and Motion similarity in Vehicle Ad Hoc Network. Proceedings of the 2024 6th International Conference on Communications, Information System and Computer Engineering (CISCE).

[27] Ferreira J., Freire M., Cruz E., Prazeres C., Figueiredo G., Peixoto M. (2025). Leading the Way: Reducing network traffic in vehicular Ad Hoc networks through cluster leader algorithms. Ad Hoc Networks.

[28] Liu G., Qi N., Chen J., Dong C., Huang Z. (2020). Enhancing clustering stability in VANET: A spectral clustering based approach. China Commun..

[29] Hu H., Lee M.J. Graph Neural Network-based Clustering Enhancement in VANET for Cooperative Driving. Proceedings of the 2022 International Conference on Artificial Intelligence in Information and Communication (ICAIIC).

[30] Dhakad B., Mishra S., Ojha S.S., Sharma J.K., Kumar S., Alrashoud M., Giri J., Hasnain S.M.M. (2024). Dynamic clustering based risk aware congestion control technique for vehicular network. Sci. Rep..

[31] Alrubaye J.S., Ghahfarokhi B.S. (2023). Resource-aware DBSCAN-based re-clustering in hybrid C-V2X/DSRC vehicular networks. PLOS ONE.

[32] Hajlaoui R., Alazmi M., Spies F. Towards Green Communication in VANETs: A Metaheuristic-Based Clustering Framework. Proceedings of the 2025 International Wireless Communications and Mobile Computing (IWCMC).

